# Symptom profiles and health-related quality of life in Korean adults with post-acute sequelae of SARS-CoV-2 infection (PASC): A latent profile analysis

**DOI:** 10.1371/journal.pone.0351506

**Published:** 2026-06-16

**Authors:** Su-jin Kim, Jinhee Kim

**Affiliations:** 1 Department of Nursing, Kwangju Women’s University, Gwangju, Republic of Korea; 2 Department of Nursing, College of Medicine, Chosun University, Gwangju, Republic of Korea; Shanghai Jiao Tong University School of Medicine, CHINA

## Abstract

**Background:**

Post-Acute Sequelae of severe acute respiratory syndrome coronavirus 2 (SARS-CoV-2) infection (PASC) is characterized by persistent and heterogeneous symptoms that impair health-related quality of life (HRQoL). Although several studies have identified symptom subgroups in Western populations using person-centered approaches, data on Asian populations remain limited.

**Objectives:**

In this study, we aimed to classify the symptom profiles of Korean adults with PASC using latent profile analysis (LPA) and examine the differences in HRQoL and associated factors between the identified profiles.

**Methods:**

We conducted an online survey of 629 adults in Korea who experienced persistent symptoms ≥12 weeks after coronavirus disease (COVID-19) diagnosis. Symptom burden was assessed using the Long COVID Symptom Tool (26 items), and HRQoL was measured using the SF-36 v2®. LPA was performed to identify the symptom subgroups. One-way analysis of variance (ANOVA) and multiple linear regression were used to compare HRQoL across profiles and explore predictors.

**Results:**

A four-class model provided the best fit: Class 1 (Low symptom, 23.3%), Class 2 (Moderate multisystem, 44.1%), Class 3 (Fatigue/post-exertional malaise dominant, 15.9%), and Class 4 (High multisystem burden, 16.7%). HRQoL differed significantly between classes (*p* < .001), with a clear gradient of decreasing scores from low to high symptom burden. The independent predictors of lower HRQoL included lower education, presence of chronic disease, poor subjective health, hospitalization during acute infection, and prolonged symptom persistence. The model explained 32.9% of the variance in HRQoL.

**Conclusions:**

Korean adults with PASC exhibit heterogeneous symptom patterns that substantially affect their HRQoL. The identification of distinct symptom profiles supports the need for tailored interventions, including rehabilitation, cognitive training, and psychological support. Our findings provide crucial evidence for developing Korean population-specific screening tools and management guidelines for PASC.

## Introduction

The World Health Organization (WHO) defined Post-Acute Sequelae of severe acute respiratory syndrome coronavirus 2 (SARS-CoV-2) infection (PASC) in 2021 as “symptoms that begin within 3 months of the onset of coronavirus disease (COVID-19) infection, last for at least 2 months, and cannot be explained by an alternative diagnosis” [1]. The U.S. Centers for Disease Control and Prevention (CDC) described a wide range of persistent, recurrent, or worsening symptoms beyond 3 months after diagnosis as Post-COVID Conditions, whereas the United Kingdom’s National Institute for Health and Care Excellence (NICE) referred to symptoms that persist beyond 12 weeks as Post-COVID-19 syndrome [2,3]. Although these definitions collectively include the sequelae that persist after the acute phase of infection, differences in diagnostic criteria, duration, and terminology make it difficult to compare study results and integrate clinical guidelines. Therefore, the term PASC, encompassing the definitions of all three institutions, has been widely adopted in academic contexts.

The estimated global prevalence of PASC among adults is approximately 10%, with more than 65 million cumulative cases reported worldwide [4]. In South Korea, as of January 2025, the Korea Disease Control and Prevention Agency (KDCA) reported approximately 16 million confirmed cases of COVID-19. Based on the WHO-estimated prevalence of 10%, more than one million individuals are presumed to have PASC [5–7]. Representative population-based studies showed that 19.7–24.9% of women and 12.7% of men experienced PASC according to the WHO criteria [5,6].

These sequelae manifest in more than 200 symptoms, including fatigue, dyspnea, cognitive impairment, arthralgia, depression, and anxiety, and may involve multiorgan dysfunction and autonomic dysregulation, leading to prolonged limitations in daily functioning [8–10]. A recent analysis of the Korean national health insurance claims data reported a substantial increase in overall medical expenditures during the COVID-19 pandemic [11]. Furthermore, a cohort study based on the United Kingdom’s OpenSAFELY platform estimated that the annual primary care cost per patient with long-term post-COVID sequelae was £2.440, with a total national expenditure of £23.38 million (approximately 3.87 billion KRW), suggesting a considerable economic burden associated with PASC management [12]. Another study reported that PASC was associated with a greater decline in health-related quality of life (HRQoL) than that observed in patients with heart failure or multiple sclerosis, highlighting its significant personal and societal impact [13].

PASC symptoms vary in onset, intensity, and duration among individuals, and multiple symptoms often co-occur to form distinct symptom clusters [14]. Variable-centered approaches are limited in capturing such heterogeneous patterns. Recent studies have applied person-centered methods, such as latent class analysis (LCA) and latent profile analysis (LPA), to demonstrate that the PASC is not a single homogeneous condition [14]. For example, four symptom profiles were identified in a U.S. cohort and five in a longitudinal study conducted in the United Kingdom, with clusters characterized as fatigue–cognitive, respiratory, and pain-dominant types [15,16]. However, most of these studies focused on Western populations, and few simultaneously examined symptom typology and HRQoL. In Korea, research on PASC symptom subtypes remains scarce, resulting in scant evidence on the characteristics of the Korean population. As HRQoL comprehensively reflects patients’ experiences and functional impairments and serves as a key basis for healthcare resource allocation, studies investigating HRQoL differences across PASC symptom profiles are urgently needed.

In this study, we aimed to (1) identify distinct symptom profiles of PASC among Korean adult COVID-19 survivors using LPA, and (2) examine differences in HRQoL and associated factors across the identified profiles. The findings are expected to provide empirical evidence for developing tailored management strategies for PASC and serve as a practical foundation for establishing Korean population-specific PASC assessment and management guidelines, as well as related health policies.

## Materials and methods

### Study design

This descriptive cross-sectional study was conducted to classify latent symptom profiles based on PASC symptoms among Korean adult COVID-19 survivors using LPA, and to identify the differences and influencing factors of HRQoL across the identified profiles.

### Participants

The study population consisted of Korean adults who had recovered from COVID-19 and were clinically diagnosed with SARS-CoV-2 infection at a medical institution, with at least one symptom persisting for > 12 weeks after the initial infection [2,3]. Initially, 880 individuals participated in the online survey. Of them, 230 were excluded for not meeting the inclusion criteria, such as missing information regarding the date of COVID-19 diagnosis, confirmation based solely on self-testing, or having post-COVID symptoms lasting < 12 weeks. Among the remaining 650 participants, responses were further screened for data quality, and cases showing careless or inconsistent responding were excluded. Specifically, exclusion criteria included (1) highly repetitive response patterns across multiple items and (2) logical inconsistencies between related variables, such as discrepancies in COVID-19 diagnosis, symptom duration, or post-COVID symptom reporting. Based on this screening process, 21 additional cases were excluded, resulting in a final sample of 629 participants.

There is no universal rule for determining the sample size required for LPA; however, simulation studies suggest that when the number of latent classes ranges from four to six, a sample size exceeding 500 generally ensures model stability and classification accuracy [17,18]. Therefore, the final sample of 629 participants was considered adequate for LPA.

### Measures

#### General characteristics and pre–COVID-19 health status.

The participants’ general characteristics included age, sex, occupation, educational level, marital status, alcohol consumption, and smoking behavior. Each item was assessed using a self-administered checklist. Pre–COVID-19 health status was evaluated based on the presence of chronic diseases and the participants’ subjective perception of health. Chronic disease status was determined using a checklist of nine chronic conditions, including hypertension, diabetes mellitus, cancer, and stroke. Participants who reported at least one condition were classified as having a chronic disease. Subjective health status was assessed using a two-point Likert scale (“healthy” vs. “unhealthy”)

#### COVID-19–related characteristics.

COVID-19–related characteristics included the number of COVID-19 vaccinations received, number of confirmed infections, whether participants visited a hospital for treatment during the acute phase, the presence of a caregiver during the infection, and the time point at which symptoms resolved after diagnosis.

#### PASC symptoms.

PASC symptoms were assessed using the *Long COVID Symptom Tool (LCST)* developed by Jeong [19], with permission from the author. This instrument consists of 26 items covering symptoms, such as fatigue, cough, difficulty concentrating, and anxiety. Each item was rated on a six-point Likert scale ranging from 0 (“no symptoms”) to 5 (“extremely severe symptoms”). The sex-specific item “menstrual cycle changes” was analyzed only among female respondents. The overall symptom severity score was calculated as the mean of all items by dividing the total score by the number of symptoms, with higher scores indicating greater overall symptom burden. For male participants, the sex-specific item was not applicable and was excluded from the calculation; therefore, the mean score was computed based on the number of available items for each individual.

#### Health-related quality of life (HRQoL).

HRQoL was assessed using the validated Korean version of the *Medical Outcomes Study 36-Item Short Form Health Survey Version II (SF-36 v2®)* developed by Ware and Sherbourne [20,21]. The official license for use was obtained from QualityMetric Incorporated (license no. Quo-03539-Y1P6WO), and scoring was performed using the *SF-36 v2® Health Outcomes™ Scoring Software* [22].

The SF-36 consists of 36 items that yield two summary scores: the *Physical Component Summary (PCS)* and the *Mental Component Summary (MCS)*. The PCS includes 10 items on physical functioning (PF), 4 items on role limitations due to physical problems (RP), 5 items on general health perceptions (GH), and 2 items on bodily pain (BP). The MCS includes 4 items on vitality (VT), 2 on social functioning (SF), 3 on role limitations due to emotional problems (RE), and 5 on mental health (MH).

In this study, the eight domain scores were converted to a 0–100 scale and the mean of these converted scores was used as a single HRQoL index. Higher scores indicated better HRQoL.

### Data collection

Data were collected from November 1 to December 31, 2023. Participants were recruited from two major online COVID-19 communities hosted on Naver, the largest internet portal in South Korea: Long COVID 119 (1,109 members) and Coronara (40,676 members). Before recruitment, official approval to post the study announcement was obtained from the administrators of both communities. A detailed notice describing the purpose of the study, participation procedures, and ethical safeguards was posted on the communities’ main announcement boards and pinned to ensure continuous visibility during the recruitment period.

Individuals who wished to participate accessed the survey through a link provided in an announcement (Google Forms). On the first page, participants were presented with a study information sheet and an online informed consent form; only those who selected “Agree” were able to proceed to the main questionnaire.

### Ethical considerations

This study was approved by the Institutional Review Board (IRB) of Chosun University (Approval no. 2–1041055-AB-N-01-2023-45) prior to data collection. Before they could access the survey link, participants were provided with a study information sheet and an online informed consent form describing the study purpose, estimated duration, potential risks and benefits, data protection measures, and the right to voluntary participation and withdrawal. Only participants who selected “I agree” could proceed to the main questionnaire.

All responses were collected anonymously without any identifiable information, such as IP addresses, and were securely stored in an encrypted research-dedicated database. Access to the dataset was restricted to the research team. The data will be retained for 3 years after study completion and then permanently deleted. A small token of appreciation was given to participants upon completion of the survey. This study was conducted in accordance with the principles of the Declaration of Helsinki and Personal Information Protection Act of the Republic of Korea.

### Data analysis

All statistical analyses were performed using SPSS Statistics (version 26.0; IBM Corp., Armonk, NY, USA) and Mplus version 8.0 (Muthén & Muthén, Los Angeles, CA, USA). Descriptive statistics were used to summarize the participants’ general and COVID-19–related characteristics and the distribution of PASC symptoms, using frequencies and percentages for categorical variables and means with standard deviations for continuous variables.

LPA was performed using 26 PASC symptom items by applying the maximum likelihood estimation with robust standard errors (MLR). Latent profile analysis was conducted using individual item responses rather than aggregated summary scores. Models with one to six latent classes were sequentially tested. Model selection was based on the following criteria: the lowest information criteria values (Akaike Information Criterion [AIC], Bayesian Information Criterion [BIC], and sample-size adjusted BIC (saBIC), a significant Lo–Mendell–Rubin adjusted likelihood ratio test (LMR-LRT) and bootstrap likelihood ratio test (BLRT) with *p* < 0.05, an entropy value ≥ 0.80, and a minimum class size of 10% of the total sample.

Differences in HRQoL across latent classes were examined using one-way analysis of variance (ANOVA). Multiple linear regression analysis was then performed to identify factors associated with HRQoL, with HRQoL as the dependent variable, and independent variables including dummy variables for latent class membership and participant characteristics that showed significant associations in the preliminary univariate analyses (*p* < 0.05).

All analyses were conducted using complete data without missing values, and two-tailed tests were used with a significance level of *p* < 0.05.

## Results

### General characteristics of the participants and COVID-19–related factors

The mean age of the 629 participants was 36.25 ± 9.62 years; participants in their 30s (42.0%) comprised the largest proportion, followed by those aged ≤ 29 years (25.1%) and those in their 40s (23.4%), while only 3.2% of the participants were aged ≥ 60 years. Women comprised 64.4% of the participants. Regarding employment status, 76.3% were currently employed and 74.4% had completed a college degree or higher. A total of 26.4% reported following a religion and 55.5% were unmarried.

In terms of health behaviors, drinking alcohol 2–4 times per month was the most common (35.3%), followed by never drinking (29.3%), and drinking less than once per month (21.6%). Non-smokers and current smokers accounted for 63.8% and 12.6% of the participants, respectively. Before COVID-19 infection, 11.4% had at least one chronic disease, while 84.9% rated their subjective health status as “healthy.”

Regarding COVID-19–related factors, 41.7% of the participants had received two vaccine doses, followed by 32.9% with three doses, and 11.0% with four or more doses, while 6.5% were unvaccinated. Most participants (77.6%) were diagnosed with COVID-19 once, 20.0% twice, and 2.4% were diagnosed three or more times. A total of 15.7% reported visiting a hospital for treatment during the acute phase and 41.5% had a caregiver during the infection. At the time of the survey, 43.4% reported that their symptoms were still persistent, 38.8% had symptoms resolve within 13–24 weeks, and 11.8% within 6–9 months (Table 1).

**Table 1:** General characteristics of the participants and COVID-19–related factors (N = 629).

Variables	Categories	N (Mean±SD)	%
Age (yrs)		(36.25 ± 9.62)	
	≤29	158	25.1
	30s	264	42.0
	40s	147	23.4
	50s	40	6.4
	≥ 60	20	3.2
Sex	Female	405	64.4
	Male	224	35.6
Employment status	Unemployed	149	23.7
	Employed	480	76.3
Level of education	Middle-school graduate or below	14	2.2
	High-school graduate	147	23.4
	≥ College	468	74.4
Religion	Yes	166	26.4
	No	463	73.6
Presence of a spouse	Yes	280	44.5
	No	349	55.5
Drinking	Never drinks	184	29.3
	Once per month	136	21.6
	2–4 times per month	222	35.3
	2–3 times per week	68	10.9
	≥ 4 times per week	19	3.0
Smoking	Non-smoker	401	63.8
	Ex-smoker	149	23.7
	Current smoker	79	12.6
Pre-existing chronic disease (before COVID-19 diagnosis)	Yes	72	11.4
	No	557	88.6
Subjective health status (before COVID-19 diagnosis)	Healthy	534	84.9
	Unhealthy	95	15.1
Number of COVID-19 vaccine doses	Unvaccinated	41	6.5
	1 dose	50	7.9
	2 doses	262	41.7
	3 doses	207	32.9
	≥ 4 doses	69	11.0
Number of confirmed COVID-19 infections	1 episode	488	77.6
	2 episodes	126	20.0
	≥ 3 episodes	15	2.4
Hospital visit for COVID-19 treatment	Yes	99	15.7
	No	530	84.3
Presence of a caregiver during infection	Yes	261	41.5
	No	368	58.5
Time to symptom resolution after COVID-19 diagnosis	13–24 weeks	244	38.8
	6–9 months	74	11.8
	9–12 months	29	4.6
	1–2 years	9	1.4
	Symptoms still present	273	43.4

N = valid responses; SD = standard deviation; yrs = years.

### Frequency and severity of PASC symptoms

Table 2 presents the frequency (percentage) and severity (mean ± standard deviation) of the 26 PASC symptoms. The most frequently reported symptoms, experienced by > 85% of the participants, were fatigue (90.6%), cough (86.3%), and post-exertional malaise (PEM) (85.2%). Other commonly reported symptoms included sore throat (81.1%), shortness of breath (dyspnea) (79.8%), sleep disturbance (79.5%), cognitive impairment (78.7%), gustatory dysfunction (loss of taste) (78.5%), fever (78.2%), and palpitations (78.2%). The least frequently reported symptom was nausea, which was experienced by 73.9% of the participants (Table 2).

**Table 2:** Descriptive statistics of post-acute sequelae of SARS-CoV-2 infection (PASC) symptoms (N = 629).

Categories	N	%	Mean±SD
Fatigue	570	90.6	3.22 ± 1.19
Post-exertional malaise	536	85.2	3.08 ± 1.23
Fever	492	78.2	2.11 ± 1.25
Shortness of breath (dyspnea)	502	79.8	2.24 ± 1.25
Cough	543	86.3	2.82 ± 1.29
Sore throat	510	81.1	2.53 ± 1.25
Chest pain	488	77.6	1.99 ± 1.16
Palpitations	492	78.2	2.01 ± 1.17
Cognitive impairment	495	78.7	2.46 ± 1.31
Headache	490	77.9	2.43 ± 1.33
Sleep disturbance	500	79.5	2.24 ± 1.30
Dizziness	488	77.6	2.18 ± 1.28
Paresthesia (numbness/tingling)	483	76.8	1.92 ± 1.19
Olfactory dysfunction (loss of smell)	484	76.9	2.39 ± 1.36
Gustatory dysfunction (loss of taste)	494	78.5	2.39 ± 1.33
Nausea	465	73.9	1.64 ± 0.97
Diarrhea	469	74.6	1.74 ± 1.07
Abdominal pain	467	74.2	1.70 ± 1.04
Loss of appetite	477	75.8	2.11 ± 1.26
Arthralgia (joint pain)	478	76.0	1.94 ± 1.18
Myalgia (muscle pain)	473	75.2	2.09 ± 1.24
Skin rash	469	74.6	1.78 ± 1.13
Hair loss	471	74.9	1.76 ± 1.15
Menstrual-cycle changes	472	75.0	1.86 ± 1.19
Depression	489	77.7	2.13 ± 1.25
Anxiety	485	77.1	2.19 ± 1.27

SD = mean ± standard deviation; N = symptom present.

Regarding symptom severity, fatigue (3.22 ± 1.19) and PEM (3.08 ± 1.23) had the highest mean severity scores, followed by cough (2.82 ± 1.29), sore throat (2.53 ± 1.25), cognitive impairment (2.46 ± 1.31), headache (2.43 ± 1.33), olfactory dysfunction (loss of smell) (2.39 ± 1.36), and gustatory dysfunction (loss of taste) (2.39 ± 1.33). Nausea had the lowest mean severity (1.64 ± 0.97) (Table 2).

### Model-fit comparison of latent profile models

Table 3 presents the model-fit indices for the latent profile models with two to six classes (k = 2–6). The information criteria (AIC, BIC, and saBIC) consistently decreased as the number of classes increased; however, the rate of reduction diminished beyond the four-class model (ΔAIC = 453.48 from four- to five-class; ΔAIC = 324.47 from five- to six-class), indicating convergence. The BIC and saBIC values exhibited similar trends.

**Table 3:** Model-fit indices for latent profile analysis (LPA) solutions.

Classification criteria	2-class	3-class	4-class	5-class	6-class
Information criteria					
AIC	50,360.96	47,607.84	**46,871.03**	46,417.55	46,093.08
BIC	50,712.17	48,079.09	**47,462.31**	47,128.86	46,924.43
saBIC	50,461.35	47,742.55	**47,040.05**	46,620.88	46,330.73
Model comparison tests					
LMR-LRT (p)	<.001	<.001	**.035**	.126	.153
BLRT (p)	<.001	<.001	**<.001**	<.001	<.001
Classification quality					
Entropy	.98	.98	**.97**	.97	.97
Class proportions (%)					
Class 1	67.0	23.3	**23.3**	23.5	23.5
Class 2	33.0	48.4	**44.1**	42.9	13.8
Class 3		28.3	**15.9**	14.8	40.7
Class 4			**16.7**	2.4	15.2
Class 5				16.5	2.4
Class 6					4.3

Bold values indicate the preferred 4-class solution based on the lowest information-criterion values and acceptable entropy.

AIC = Akaike Information Criterion; BIC = Bayesian Information Criterion; saBIC = sample-size-adjusted BIC; LMR-LRT = Lo-Mendell-Rubin adjusted likelihood-ratio test; BLRT = bootstrap likelihood-ratio test.

Regarding the model comparison tests, BLRT was significant across all models, whereas the LMR-LRT reached statistical significance only for the four-class model (*p* = 0.035), suggesting no further improvement with additional classes. Entropy values, indicating classification accuracy, were high for all models (≥ 0.97), with the four-class model also demonstrating an entropy of 0.97.

In the four-class model, the class proportions were 23.3% (Class 1), 44.1% (Class 2), 15.9% (Class 3), and 16.7% (Class 4), satisfying the criterion of at least 10% per class and indicating no imbalance in class size. In contrast, models with five or more classes produced very small groups (2.4%).

Considering the change in information criteria, the significance of LMR-LRT, high entropy values, stable class proportions, and interpretability, the four-class model was determined to be the optimal solution (Table 3).

### Characteristics of latent PASC symptom profiles

The four-class model derived from the LPA was identified as the optimal solution. The characteristics of each latent class are as follows.

Class 1 (n = 146, 23.3%) showed the lowest mean symptom severity score (0.38 ± 0.26) among the four groups and was labeled the low-symptom profile.

Class 2 (n = 278, 44.1%) demonstrated a mean symptom severity score of 1.55 ± 0.27, which was higher than that of Class 1 but lower than those of Classes 3 and 4. This group was labeled as having a moderate multisystem profile.

Class 3 (n = 105, 15.9%) had a mean severity score of 2.59 ± 0.32, higher than those of Classes 1 and 2 but lower than that of Class 4, indicating a high-symptom group. This class showed the highest scores for fatigue and PEM (3.78 ± 0.96) and was labeled the fatigue/PEM-dominant profile.

**Fig 1:**
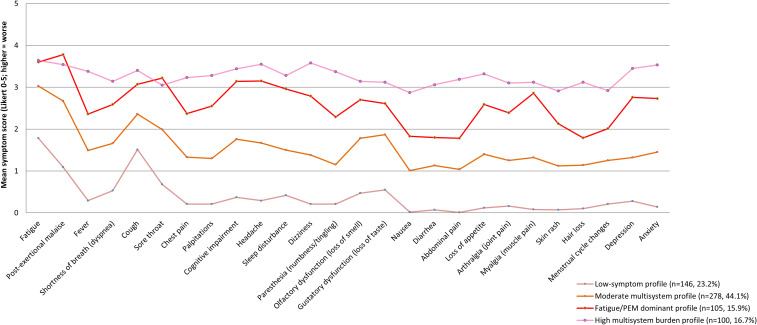
PASC symptom severity across four latent profiles (K = 4). Note. Mean scores for 26 symptoms are plotted on a 0–5 Likert scale (higher scores indicate worse symptoms). Profiles are ordered by overall severity: Low-symptom, Moderate multisystem, Fatigue/PEM-dominant, and High multisystem burden. The sex-specific item (‘Menstrual cycle changes’) was calculated among female respondents only. PEM = post-exertional malaise.

Class 4 (n = 100, 16.7%) exhibited the highest overall mean severity score (3.25 ± 0.42). Most of the 26 symptoms in this class had mean scores of ≥ 3.0, indicating a substantial symptom burden. Accordingly, this group was labeled as having a high multisystem burden profile (Fig 1).

### Differences in HRQoL

The mean HRQoL score among the participants was 70.79 ± 8.53. Statistically significant differences in HRQoL were observed across age, education level, smoking status, pre-existing chronic disease, subjective health status, number of COVID-19 vaccine doses, hospital visits for acute COVID-19 treatment, time to symptom resolution after diagnosis, and latent symptom profile class.

By age group, participants aged ≤ 29 years showed the highest mean HRQoL (72.92 ± 5.63), followed by those in their 40s (71.03 ± 7.85), whereas those in their 50s had the lowest HRQoL (68.97 ± 7.80; *p* = .003). Regarding education level, participants with a college degree or higher reported significantly higher HRQoL (71.87 ± 7.38) than those with middle school education or lower (62.86 ± 9.01; *p* < .001).

In terms of smoking status, non-smokers had the highest HRQoL (72.00 ± 7.97), followed by current smokers (70.78 ± 7.58) and former smokers (67.54 ± 9.62; *p* < .001). Participants with pre-existing chronic diseases reported lower HRQoL (65.63 ± 10.02) than those without (71.46 ± 8.09; *p* < .001). Similarly, those who perceived their health as “healthy” (71.68 ± 8.19) had significantly higher HRQoL than those who perceived themselves as “unhealthy” (65.81 ± 8.74; *p* < .001).

With regard to vaccination status, HRQoL differed significantly by the number of doses received: participants with four or more doses had the lowest HRQoL (66.38 ± 11.37), followed by unvaccinated individuals (68.05 ± 9.60), those with one dose (69.74 ± 7.39), two doses (71.27 ± 7.42), and three doses (72.45 ± 8.20; *p* < .001). Participants who visited a hospital for acute COVID-19 treatment reported lower HRQoL (66.63 ± 8.88) than those who did not (71.76 ± 8.12; *p* < .001).

Regarding symptom resolution period, participants whose symptoms resolved within 13–24 weeks after diagnosis had the highest HRQoL (74.40 ± 6.31), whereas those whose symptoms persisted at the time of the survey had the lowest HRQoL (67.69 ± 8.73; *p* < .001).

Across the latent symptom-profile classes, mean HRQoL scores were as follows: Class 1 (low-symptom profile), 71.68 ± 9.09; Class 2 (moderate multisystem profile), 72.87 ± 8.12; Class 3 (fatigue/PEM-dominant profile), 68.42 ± 8.88; and Class 4 (high multisystem burden profile), 66.21 ± 5.76 (*p* < .001). The HRQoL showed a stepwise decline with increasing symptom burden (Table 4).

**Table 4:** Differences in health-related quality of life by participant characteristics and latent profile classes (N = 629).

Variables	Categories	Mean±SD	t/F	*p*
SF-36		70.79 ± 8.53		
Age (yrs)	≤ 29	72.92 ± 5.63	4.10	.003
	30s	69.72 ± 10.17		
	40s	71.03 ± 7.85		
	50s	68.97 ± 7.80		
	≥ 60	70.00 ± 7.29		
Sex	Female	70.61 ± 9.03	−7.56	.450
	Male	71.12 ± 7.55		
Employment status	Unemployed	70.85 ± 9.29	0.09	.926
	Employed	70.77 ± 8.29		
Level of education	Middle-school graduate or below	62.86 ± 9.01	18.03	<.001
	High-school graduate	68.10 ± 10.71		
	≥ College	71.87 ± 7.38		
Religion	Yes	69.77 ± 9.03	−1.73	.085
	No	71.16 ± 8.32		
Presence of a spouse	Yes	70.78 ± 8.94	−0.04	.965
	No	70.81 ± 8.20		
Drinking	Never drinks	70.27 ± 8.68	2.19	.069
	Once per month	72.51 ± 7.42		
	2–4 times per month	70.12 ± 9.18		
	2–3 times per week	70.41 ± 8.24		
	≥ 4 times per week	72.74 ± 6.27		
Smoking	Non-smoker	72.00 ± 7.97	15.55	<.001
	Ex-smoker	67.54 ± 9.62		
	Current smoker	70.78 ± 7.58		
Pre-existing chronic disease (before COVID-19 diagnosis)	Yes	65.63 ± 10.02	−5.58	<.001
	No	71.46 ± 8.09		
Subjective health status (before COVID-19 diagnosis)	Healthy	71.68 ± 8.19	6.08	<.001
	Unhealthy	65.81 ± 8.74		
Number of COVID-19 vaccine doses	Unvaccinated	68.05 ± 9.60	8.40	<.001
	1 dose	69.74 ± 7.39		
	2 doses	71.27 ± 7.42		
	3 doses	72.45 ± 8.20		
	≥ 4 doses	66.38 ± 11.37		
Number of confirmed COVID-19 infections	1 episode	70.84 ± 8.80	0.44	.640
	2 episodes	70.85 ± 7.26		
	≥ 3 episodes	68.73 ± 9.74		
Hospital visit for COVID-19 treatment	Yes	66.63 ± 8.88	−6.38	<.001
	No	71.76 ± 8.12		
Presence of a caregiver during infection	Yes	71.06 ± 7.59	0.65	.511
	No	70.60 ± 7.15		
Time to symptom resolution after COVID-19 diagnosis	13–24 weeks	74.40 ± 6.31	23.38	<.001
	6–9 months	71.30 ± 8.21		
	9–12 months	68.62 ± 11.67		
	1–2 years	69.78 ± 8.74		
	Symptoms still present	67.69 ± 8.73		
Latent symptom-profile class	Class 1	71.68 ± 9.09	20.42	<.001
	Class 2	72.87 ± 8.12		
	Class 3	68.42 ± 8.88		
	Class 4	66.21 ± 5.76		

SD = standard deviation; yrs = years.

### Predictors of HRQoL

To identify the predictors of HRQoL, multiple linear regression analysis was performed, including the participant characteristics that showed significant differences in HRQoL in the univariate analyses (age, level of education, smoking status, pre-existing chronic disease, subjective health status, number of COVID-19 vaccine doses, hospital visits for acute COVID-19 treatment, and time to symptom resolution after diagnosis), along with the latent symptom profile class. Categorical variables were dummy coded before being included in the regression models.

The overall regression model was statistically significant (*F* = 13.50, *p* < .001), with the included independent variables explaining approximately 32.9% of the variance in HRQoL scores (*R²* = .329). The Durbin–Watson statistic was 1.69, indicating no autocorrelation of the residuals. Tolerance values ranged from 0.11 to 0.94, and variance inflation factors (VIFs) ranged from 1.05 to 9.32, confirming the absence of multicollinearity. Examination of the residual plots and standardized residuals indicated that the assumptions of linearity, homoscedasticity, and normality were satisfied.

Among the independent variables, participants with a college education or higher showed significantly higher HRQoL than those with middle school education or lower (*β* = .29, *p* = .004). HRQoL was also significantly higher among participants without pre-existing chronic diseases (*β* = .10, *p* = .008) and among those who perceived themselves as healthy (*β* = .14, *p* < .001). Conversely, participants who visited a hospital for acute COVID-19 treatment had significantly lower HRQoL (*β* = –.11, *p* = .004).

Compared with participants whose symptoms persisted at the time of the survey, those whose symptoms resolved within 13–24 weeks (*β* = .23, *p* < .001) and within 6–9 months (*β* = .10, *p* = .006) reported significantly higher HRQoL. Regarding latent symptom-profile class, participants in Class 2 (moderate multisystem profile) (*β* = –.09, *p* = .024) and Class 4 (high multisystem burden profile) (*β* = –.17, *p* < .001) had significantly lower HRQoL than those in Class 1 (low-symptom profile). Interestingly, participants in Class 3 (fatigue/PEM-dominant profile) had higher HRQoL compared with those in Class 1 (*β* = .02, *p* < .001) (Table 5). These results represent associations after adjustment for potential confounding variables, whereas the descriptive comparisons are based on unadjusted mean differences across classes.

**Table 5:** Predictors of health related quality of life (N = 629).

Variables	Categories	B	SE	95% CI	β	t	*p*
(Constant)		59.49	2.94	53.71/ 65.26		20.22	<.001
Age (yrs)	≤ 29	−0.59	1.81	−4.15/ 2.96	−.03	−0.32	.744
	30s	−1.40	1.75	−4.85/ 2.04	−.08	−0.80	.423
	40s	−0.29	1.78	−3.80/ 3.21	−.01	−0.16	.869
	50s-	−0.33	2.02	−4.30/ 3.60	−.01	−0.16	.868
	≥ 60	Ref.					
Level of education	Middle-school graduate or below	Ref.					
	High-school graduate	3.09	2.04	−0.92/ 7.12	.15	1.51	.131
	≥ College	5.80	1.98	1.91/ 9.70	.29	2.92	.004
Smoking	Non-smoker	0.42	0.90	−1.35/ 2.19	.02	0.46	.640
	Ex-smoker	−1.88	1.01	−3.87/ 0.10	−.94	−1.86	.063
	Current smoker	Ref.					
Pre-existing chronic disease (before COVID-19 diagnosis)	Yes	Ref.					
	No	2.70	1.01	0.71/ 4.68	.10	2.66	.008
Subjective health status (before COVID-19 diagnosis)	Healthy	3.42	0.87	1.70/ 5.14	.14	3.91	*<.*001
	Unhealthy	Ref.					
Number of COVID-19 vaccine doses	Unvaccinated	Ref.					
	1 dose	0.65	1.54	−2.37/ 3.68	.02	0.42	.670
	2 doses	1.90	1.22	−0.49/ 4.31	.11	1.55	.120
	3 doses	2.37	1.25	−0.08/ 4.83	.12	1.89	.059
	≥ 4 doses	−2.63	1.49	−5.56/ 0.30	−.09	−1.75	.079
Hospital visit for COVID-19 treatment	Yes	−2.60	0.90	−4.37/ −0.83	−.11	−2.89	.004
	No	Ref.					
Time to symptom resolution after COVID-19 diagnosis	13–24 weeks	3.99	0.71	2.58/ 5.39	.23	5.56	*<.*001
	6–9 months	2.77	1.00	0.79/ 4.75	.10	2.75	.006
	9–12 months	0.11	1.47	−2.77/ 3.00	.00	0.07	.938
	1–2 years	0.51	2.46	−4.05/ 5.07	−.09	0.21	.836
	Symptoms still present	Ref.					
Latent symptom-profile class	Class 1	Ref.					
	Class 2	−2.16	0.96	−4.05/ −0.28	−.09	−2.25	.024
	Class 3	3.53	0.76	2.04/ 5.02	.02	4.64	<.001
	Class 4	−4.08	1.02	−6.09/ −2.07	−.17	−3.99	*<.*001
R² = .329; Adj. R² = .305; F = 13.50; *p < .*001; Durbin-Watson = 1.69							

Ref = reference group; B = unstandardized coefficient; β = standardized coefficient; SE = standard error; CI = confidence interval; yrs = years.

## Discussion

This study is one of the first large-scale analyses in Korea to classify the symptom patterns of PASC among adults using LPA, and to examine differences in HRQoL across symptom profiles. The optimal model consisted of four distinct classes that aligned closely with the four- to five-profile structures reported in Western cohort studies [15,16]. The four identified profiles were labeled low-symptom, moderate multisystem, fatigue/PEM-dominant, and high multisystem burden profiles. Notably, the latter two groups exhibited more complex and severe manifestations, characterized by PEM, cognitive dysfunction, and pain. These findings confirm that PASC is not a single disease entity, but a heterogeneous syndrome encompassing multiple symptom domains [4,14,23].

A clear gradient was observed in the HRQoL analysis, indicating that quality of life decreased progressively with increasing symptom burden. Notably, however, although the moderate multisystem profile (Class 2) showed the highest mean HRQoL in the unadjusted comparisons, this pattern was attenuated in the regression analysis after adjustment for covariates. This discrepancy likely reflects differences in the distribution of sociodemographic and clinical characteristics across classes, such as educational attainment, subjective health status, and the burden of chronic conditions, which may independently influence HRQoL. In contrast, the fatigue/PEM-dominant profile (Class 3) exhibited relatively higher HRQoL in the adjusted model, suggesting that specific symptom patterns may be differently associated with HRQoL compared to overall symptom burden when individual characteristics are taken into account. Overall, this pattern reflects the influence of covariate adjustment, indicating that the observed differences in HRQoL across latent classes should be interpreted in the context of underlying participant characteristics rather than as simple unadjusted group differences.

Education level, presence of chronic diseases, subjective health perception, history of hospital visits for acute COVID-19 treatment, and time-to-symptom resolution were identified as independent predictors of HRQoL. The multiple regression model explained approximately 32.9% of the variance in HRQoL (*R²* = .329), which was comparable to or slightly higher than the explanatory power reported in previous studies on HRQoL among patients with PASC [13]. These findings suggest that multidimensional risk factors should be considered when developing strategies for managing PASC in Korean adults.

When compared with findings from Western studies, the four symptom profiles identified in this study were consistent in that they all included a high-burden group characterized by a combination of fatigue, cognitive impairment, and pain. A longitudinal study in the United Kingdom reported fatigue/cognitive-dominant and respiratory/pain-type clusters [15], whereas a large-scale data analysis in the U.S identified a high multisystem burden group [16]. The fatigue/PEM-dominant class observed in this study is consistent with the abovementioned findings and notably overlaps with the PEM commonly reported among patients with myalgic encephalomyelitis/chronic fatigue syndrome (ME/CFS) [24], supporting the potential pathophysiological continuity between PASC and ME/CFS.

However, the proportion of the low-symptom class was relatively high (23%) in the Korean sample, which was probably due to the characteristics of online recruitment. Individuals who voluntarily participate in online surveys often exhibit higher health-related anxiety, which could result in differences in the actual population distribution of low-symptom cases [6,7]. In addition, the “respiratory-dominant” phenotype commonly reported in Western studies was not clearly observed in this study. This discrepancy may be related to clinical characteristics specific to Asian populations, differences in vaccination rates, or variations in the dominant viral strains during different epidemic periods [5,9]. Therefore, nationwide population-based surveys and linkage studies incorporating clinical data are warranted.

The mean HRQoL level among the participants was significantly lower than that of the general Korean adult population and comparable to that reported in patients with heart failure or multiple sclerosis [13]. Given the relatively high prevalence of several symptoms observed in this study, these findings should be interpreted with caution. In particular, individuals with persistent symptoms showed markedly lower HRQoL scores, indicating that PASC can have substantial long-term effects on physical, mental, and social functioning [8,9,13,25]. Educational attainment emerged as an important protective factor, likely reflecting its close association with health literacy and access to resources [18,19]. Conversely, the presence of chronic disease and hospitalization during the acute phase of COVID-19 were associated with lower HRQoL, suggesting a “double burden” effect in which PASC exacerbates pre-existing conditions [10,11].

The number of COVID-19 vaccine doses was associated with HRQoL in a nonlinear pattern rather than a simple linear association, which may indicate interactions with factors such as the persistence of post-vaccination symptoms or reinfection experiences, as reported in previous studies, warranting further investigation [12]. Notably, this association should be interpreted with caution, as individuals with different vaccination histories may differ systematically in age, pre-existing health conditions, and healthcare access, all of which can independently influence HRQoL. Therefore, the observed relationship may partly reflect underlying differences in population characteristics rather than a direct effect of vaccination alone. Hospitalization during the acute phase and delayed symptom resolution were major predictors of lower HRQoL. In particular, participants whose symptoms persisted for > 6 months exhibited markedly reduced HRQoL, underscoring the urgent need to establish a long-term management system for post-COVID sequelae [4,20].

This study makes a significant academic contribution by demonstrating, using national data, that PASC is not a single disease entity, but a complex, multifaceted syndrome. A symptom profile–based approach is clinically useful because it reflects the individual symptom patterns of patients. These findings suggest the need for tailored management strategies for individuals with PASC, and provide a foundation for developing multidisciplinary intervention models that include rehabilitation, cognitive training, and psychosocial support [17,24,26]. Clinically, applying profile-based predictive models may facilitate the early identification and targeted management of high-risk groups. The fatigue/PEM-dominant group may benefit from early rehabilitation interventions, whereas the high multisystem burden group should be prioritized for integrated multisystem care [14,16]. The observed HRQoL gradient supports a model in which a higher symptom burden is associated with poorer quality of life, suggesting that even simple symptom screening in clinical settings could help identify patients at high risk of reduced HRQoL.

From a policy perspective, our findings support long-term vaccination strategies and investments in PASC rehabilitation programs. In Korea, studies have estimated that several hundred thousand individuals may experience PASC [5,7], and the absence of a long-term management system can lead to substantial social and economic costs [11,12]. Beyond the individual burden of reduced quality of life, PASC is also associated with productivity loss and increased healthcare expenditure. The United Kingdom’s OpenSAFELY study reported higher annual primary care costs among patients with long-term sequelae [12,13], a finding that may serve as a relevant policy reference in Korea. Therefore, early intervention and optimized resource allocation are essential for reducing the cost burden.

When developing national PASC management guidelines, it is crucial to incorporate tailored management strategies based on symptom profiles. Management approaches that distinguish between the low- and high-symptom groups can enhance policy efficiency and clinical effectiveness. Furthermore, the inclusion of PASC-related support items in the national health insurance system and the expansion of community-based rehabilitation and psychosocial support services are urgently warranted [21,22].

This study is the first in Korea to apply LPA for classifying PASC symptom patterns using a large sample of COVID-19 survivors (n = 629). In particular, 26 symptom domains and HRQoL were simultaneously assessed using the SF-36 v2® instrument, allowing for a multidimensional analysis of the association between symptom burden and HRQoL. The use of an officially licensed assessment tool and a high entropy value (>.97) support the methodological rigour and classification accuracy of the latent classes identified in this study.

Nevertheless, this study has several limitations. First, because this study employed a cross-sectional design, causal relationships between latent symptom profiles and HRQoL could not be determined. Second, the participants were recruited through online communities, which may have led to an overrepresentation of individuals with more severe symptoms, resulting in potential selection bias. Therefore, caution is warranted when generalizing these findings to the broader population of individuals with PASC. Third, major variables, including symptom experience, vaccination, and hospitalization, were based on self-reported data, which may have introduced recall and information biases. Fourth, objective biological indicators (such as laboratory or imaging findings) were not included, limiting the ability to directly examine the association between symptom profiles and underlying physiological mechanisms. Fifth, HRQoL was calculated as a single composite index by averaging the eight domain scores of the SF-36 v2® after conversion to a 0–100 scale. However, because the SF-36 is inherently a multidimensional instrument, reporting domain-specific scores and summary measures (*PCS/MCS*) is generally recommended, and the validity of a single total score remains unestablished [20–22]. Therefore, potential limitations in construct validity, as well as dilution of domain-specific effects, should be considered when interpreting the findings of this study. In particular, the use of a composite score may have obscured differential patterns between physical and mental components of HRQoL across symptom profiles. Future studies should incorporate both domain-specific scores and standardized summary measures (PCS, MCS, and norm-based scores) to provide a more comprehensive and interpretable assessment of HRQoL and to enhance comparability across studies.

However, this study is significant because it identified the heterogeneity of PASC symptom profiles and differences in HRQoL among Korean adults using a large-scale dataset. Future research should adopt a longitudinal design to track changes in symptom profiles and HRQoL trajectories over time. In particular, investigating the relationships between objective biomarkers (such as autonomic nervous system indices and inflammatory markers) and symptom profiles could help elucidate the underlying biological mechanisms. Additionally, randomized controlled trials applying tailored interventions, such as exercise, cognitive-behavioral therapy, and psychological support, according to symptom profiles, are warranted. Finally, expanding the research to include children, older adults, and vulnerable populations will enhance the comprehensiveness of future policy recommendations [27].

In conclusion, this study identified four heterogeneous symptom profiles among Korean adults with PASC and demonstrated the independent effects of profile-specific clinical characteristics on HRQoL. These findings provide fundamental evidence for the development of Korean population-specific PASC assessment tools and the establishment of tailored management strategies and health policies.

## Supporting information

S1 Data Anonymized dataset used in the latent profile analysis of symptom profiles and health-related quality of life among Korean adults with post-acute sequelae of SARS-CoV-2 infection (PASC). (XLSX)
